# Action ethical dilemmas in surgery: an interview study of practicing surgeons

**DOI:** 10.1186/1472-6939-6-7

**Published:** 2005-07-04

**Authors:** Kirsti Torjuul, Ann Nordam, Venke Sørlie

**Affiliations:** 1 Sør-Trøndelag University College, Faculty of Nursing, Trondheim, Norway; 2 Centre for Medical Ethics, University of Oslo, Norway; 3 Institute of Nursing and Health Sciences, Faculty of Medicine, University of Oslo, Norway

## Abstract

**Background:**

The aim of this study was to describe the kinds of ethical dilemmas surgeons face during practice.

**Methods:**

Five male and five female surgeons at a University hospital in Norway were interviewed as part of a comprehensive investigation into the narratives of physicians and nurses about ethically difficult situations in surgical units. The transcribed interview texts were subjected to a phenomenological-hermeneutic interpretation.

**Results:**

No gender differences were found in the kinds of ethical dilemmas identified among male and female surgeons. The main finding was that surgeons experienced ethical dilemmas in deciding the right treatment in different situations. The dilemmas included starting or withholding treatment, continuing or withdrawing treatment, overtreatment, respecting the patients and meeting patients' expectations. The main focus in the narratives was on ethical dilemmas concerning the patients' well-being, treatment and care. The surgeons narrated about whether they should act according to their own convictions or according to the opinions of principal colleagues or colleagues from other departments. Handling incompetent colleagues was also seen as an ethical dilemma. Prioritization of limited resources and following social laws and regulations represented ethical dilemmas when they contradicted what the surgeons considered was in the patients' best interests.

**Conclusion:**

The surgeons seemed confident in their professional role although the many ethical dilemmas they experienced in trying to meet the expectations of patients, colleagues and society also made them professionally and personally vulnerable.

## Background

Surgeons are responsible for all activities related to patients' treatment and care in surgical units and it is therefore important for them to act in a the best and correct way towards patients, relatives, and colleagues. Studies have shown, however, that physicians often are in doubt about the best and correct actions to take for the patients in specific situations [1-3]. This question is not only a medical one, but can be understood in both action and relational ethical perspectives. A relational ethical perspective means reflecting on the challenges we encounter in our relationships with others and how to best fulfil our social roles and obligations – as a person, a surgeon, and a colleague. It tries to answer questions such as "How can I adequately meet the challenges that confront me in the relationships in which I am involved in this situation?" [4]. Qualities that make a person a good physician are not only individual traits but they are characteristics of the relationships [5].

Surgeons' responsibility and the imperative to save life often lead to their focusing on an action ethical perspective in their reasoning [1,4]. Reasoning according to this perspective means explaining our choices of actions in situations in which we are not sure what the right thing to do is. In this perspective, ethics often centers on difficult ethical dilemmas and decision-making, justifying our actions, and gives answers to the questions: What should I (we) do? Did I (we) do the right thing in this situation? [4]. Ethical dilemmas occur when physicians have to choose between at least two alternative and equally difficult courses of actions. Because neither of the alternatives have positive outcomes, physicians have to choose between two evils [4]. Ethical dilemmas can also be understood as conflicts between different courses of actions that result from following general and mutually exclusive ethical principles in medicine [6].

Action and relational ethical perspectives are not mutually exclusive, but rather complementary, as surgeons have a dual responsibility for their actions in specific situations as well as their way of being in their relationships [4,7]. Being a good surgeon presupposes both professional competencies based on scientific and clinical knowledge and skills, and being present and showing respect and compassion for patients [4,5,8].

Surgeons today are confronted with more ethical dilemmas than before due to the growth in scientific knowledge, an increase in the availability and efficiency of medical technology, a more equal relationship between patients and surgeons, and changes in the organizational arrangement and financing of the health care system [3,9,10]. The growth in scientific knowledge and technology has given surgeons new and better diagnostic equipment and treatment opportunities. The frequency of surgical treatment is expanding and surgeons are able to successfully operate older patients and patients with multiple and more serious diseases than before [3,11]. New treatment opportunities have increased the number of possible ethical dilemmas in surgical practice and put heavy pressure on the individual surgeon who has a personal responsibility for all decisions concerning the patients' treatment and care. Ethical considerations cannot be avoided when surgeons have to choose between what ought to be done among the many courses of action that are available for patients in particular situations [10]. Physicians and surgeons are said to experience a decrease in their autonomy at the same time because more external factors and stakeholders are influencing their decisions concerning patients' treatment [3,10,12].

The development of better anaesthetic methods and less invasive surgical techniques has made it possible to increase the frequency of surgery and perform rather extensive operations in out-patient facilities. The length of patients' stays in hospitals has also been reduced. The growth of new diagnostic and therapeutic opportunities in modern medicine have, in turn, created great demands on resources and made medicine a high cost endeavor. Economic factors are said to increasingly determine the patterns of clinical work, either directly or indirectly, and physicians frequently experience the ethical dilemma of allocating limited resources [13]. It is argued that surgeons have been put under heavy political and administrative pressure to reduce costs to a greater extent than other medical specialists, and they may experience dilemmas between promoting the patients' health interests and the economic interest of the hospital and of society [3,14].

Patients today are said to expect more from medical diagnostics and treatment than before, expectations that may be greater than physicians are able to provide [15]. They almost take for granted that everything can be treated and cured and are more willing to sue physicians for suboptimal results of treatment or deviation from perfect performance [9,16]. Surgeons often experience high expectations from patients, patients' relatives, colleagues and the media, and can even feel pressure to perform innovative and undocumented surgical operations [3,11,17-19]. The fear of being sued can lead to defensive medicine and reduced trust between physicians and their patients [20]. Health care is increasingly perceived as a commodity [21] and consumerism may lead physicians to spend more time attending to patients' wants than before, and this has made the physicians work more complicated [13,16,22,23].

Few empirical studies have been found that explore the ethical dilemmas in surgery from the surgeons' point of view and their experiences of ethically difficult situations in during practice. The present study is part of a comprehensive investigation into the narratives of physicians and nurses about ethically difficult situations and the meaning of being in such situations in surgical units.

The results of this interview study with male and female surgeons will be presented in two papers. The present study describes the kinds of ethical dilemmas surgeons face in their practice. The other paper describes the surgeons' experiences of being in ethically difficult situations in their relationships with patients, relatives and colleagues [24]. The results from the interviews with the registered nurses (RNs) working in surgical units are in progress and will be addressed in a third paper.

## Methods

### Participants and setting

Five male and five female surgeons working in surgical units at a university hospital in Norway participated in the study. All were experienced and had been working in health care from 9 to 31 years (median = 21.5), and in surgery between 5 to 21 years (median = 13). The surgeons worked full time and were on duty when the interviews were conducted. No individual characteristics will be disclosed in order to guarantee anonymity. The surgeons gave their informed consent to participate in the study, which was approved by the 5^th ^Regional Ethics Committee in Norway.

### Data collection

#### Interviews

The interviews were conducted by the first author and lasted from 35 to 75 minutes (median = 55). They were tape recorded and subsequently transcribed verbatim. The interviewees were asked to tell about one or more ethically difficult care situations that they had experienced in their work as surgeons. What constituted an ethically difficult situation was not defined, allowing the interviewees to determine what they considered ethically difficult themselves. The aim of the interviews was to obtain as many rich narratives as possible without interrupting the surgeons' narrative flow and reflection. If the surgeons did not spontaneously reflect on the events they talked about, their reflections were sought. Questions were asked when the interviewer wanted the interviewees to elaborate on their stories or had difficulty understanding the narration. These questions referred to the interviewees' thoughts, feelings, and actions [25]. Field notes were taken during the interview as aids to the interviewer's memory and in order to understand the interview text in relation to its context, e.g. arrangements and interruptions. Nonverbal communications that seemed relevant were also noted, such as laughter and long pauses. The transcribed text was compared with the field notes and adjusted if necessary.

### Data analysis

#### Interpretations

The method of interpretation used was inspired by the French philosopher Paul Ricoeur's phenomenological hermeneutics [26], and developed at the University of Tromsø (Norway) and Umeå University (Sweden) and has previously been used by Lindseth et al., [27] Udén et al., [1,28] Søderberg and Norberg., [29] Sørlie et al., [18,30-33] and Nordam et al. [34]. This method is useful to elucidate the narratives of people's experiences. The method of interpretation proceeds through three phases, which constitute a dialectical movement between the whole and the parts of the text and between understanding and explanation [26].

Each interview was regarded as a text. First, a *naïve reading *was made of all the transcribed interviews as a whole to gain a first impression of the surgeons' experiences of ethical dilemmas in their clinical work. The repeated naïve reading was made as open-minded as possible, without any deliberate analysis of the text. The naïve reading shows the direction the structural analysis may take. Second, a *structural analysis *was performed in order to validate or refute the initial understanding obtained from the naïve reading and to explain what the text was saying. The interviews were divided into meaningful parts and patterns, i.e. one sentence, parts of a sentence, or a whole paragraph with a related meaning content. The meaning units were condensed and discussed among all the authors, and themes and subthemes were identified, and presented in 'Results'. Third, a *comprehensive understanding *was developed, taking into account the authors' pre-understanding, the naïve reading, and the structural analysis (results). The text was read as a whole and interpreted in relation to relevant theories of ethics and results from previous investigations into ethical dilemmas in surgery [35]. The comprehensive understanding is presented under the heading 'Discussion'.

The analysis was conducted by all the authors and the interpretative agreement was considered satisfactory and to be the most useful understanding of the surgeons' experiences of ethical dilemmas in their clinical work. The authors' interpretation was not shared or validated with the surgeons. A kind of validation is accomplished by the structural analysis as the objective part of the interpretation process [35].

## Results

Several readings of the interview texts revealed that the surgeons narrated about ethical dilemmas they face in their practice. They also told about the ethical challenges that confront them in their relationships with patients and colleagues and their experiences of living with these challenges. The surgeons admitted that they were faced with several ethical dilemmas at the same time, but concluded that consideration for their patients was the main single factor in resolving these dilemmas. No gender differences were found in the kinds of ethical dilemmas identified among male and female surgeons. The results showed that each surgeon created many and detailed narratives. When the surgeons were asked to tell about the ethically difficult situations that they had experienced, they did not differentiate between action and relational perspectives in their narration. This is an analytical distinction made by the authors in order to structure the results. The authors therefore decided to separate the presentation of the results in two papers: one paper about the surgeons' experiences of being in ethically difficult situations from a relational ethical perspective [24] and the other paper about the surgeons' experiences of ethically difficult situations from an action ethical perspective according to the theory presented in the background [4]. This paper presents the kinds of ethical dilemmas surgeons are faced with during practice.

The themes and the subthemes from the structural analysis are shown in Table 1 and presented in the text below. Direct quotations from the interviewers are included to illuminate the results.

**Table 1:** Overview of themes and subthemes that emerged from the structural analysis of interviews with the surgeons about ethical dilemmas.

**Themes**	**Subthemes**
**Treatment**	*Starting or withholding treatment* *Continuing or withdrawing treatment* *Overtreatment* *Respecting the patients* *Meeting patients expectations*
**Resolving differences in opinions**	*Superior colleagues* *Colleagues from other departments* *Incompetent colleagues*
**Society**	*Local limited resources* *Laws and regulations* *Global limited resources*

### Treatment

The interviews revealed that the surgeons experienced ethical dilemmas on almost a daily or weekly basis. They emphasized that decision making is strongly dependent on the context as each situation and patient is unique. Making existential decisions, like withholding or withdrawing treatment where patients' lives and quality of lives are at stake, were the main ethical dilemmas narrated by the surgeons.

#### Starting or withholding treatment

The surgeons related that they experience the dilemma of staring and withholding treatment especially when working with incurable cancer patients, very old patients with additional physical and mental diseases, and with emergency and trauma patients. In situations when the disease cannot be cured, palliative operations can cause complications, prolonged suffering and an earlier death. A newborn child with serious congenital malformations or deformities may also raise the dilemma of starting or withholding treatment. Although the malformations can be corrected so that the child's life is saved, the result may be a life with serious handicaps and a reduced quality of life for both the child and its parents.

The question of starting or withholding treatment was experienced as most dramatic when patients were admitted with life-threatening conditions like a ruptured aortic aneurysm which will lead to death without an operation, but where the outcome of surgery and the patients' quality of life afterwards are impossible to predict. "You know that if you do not do anything immediately, the patient will die. If you start you have a chance of making it. You give it a try". The surgeons said that it is more difficult to withhold treatment the younger the patients are, mainly because the emotional feelings surrounding the decisions are experienced as more difficult. Wondering about starting or withholding treatment means that the surgeons are uncertain about the outcome of surgery. The surgeons remembered cases of unexpected recovery and success against all odds. "We experience patients where everything looks hopeless and we go on doubting for a while. And then they recover from a seemingly hopeless condition and live well, even with severe complications. We have seen people with paralyzed legs, a stoma and permanent kidney failure, who are nevertheless content for having survived. This shows how difficult it is to assess a person's quality of life and how they will look at things afterwards".

The surgeons said that if there is the slightest possibility of survival, they usually try to operate in order to give the patients a chance, especially if the patients are conscious and strongly want the operation. They said that most patients are willing to undergo any treatment although their prognoses are poor. Withholding treatment was felt like "destroying all means of retreat".

#### Continuing or withdrawing treatment

The decision whether to withdraw active medical treatment other than palliative, or to continue treatment, was experienced as equally difficult by the surgeons. They said that it is more difficult to withdraw treatment the younger the patients are. Patients' expectations are important when the surgeons make their decisions. "If the patients are able to express that they do not want any more [treatment], then the decision is simple. But if they are unable to express themselves, it is very difficult to end an ongoing treatment". The surgeons said that they always consult the patients' relatives before a decision to withdraw treatment is made, but stressed that it is not the relatives that should make the final decision, but the physicians.

#### Overtreatment

Finding the right level of treatment was experienced as an ethical dilemma for severely ill and very old patients and patients with non-curable cancer. The surgeons said that they attempt to give every patient a chance to survive, and avoid the risk of termination of treatment too early may result in overtreatment, and prolonging the patients' pain and suffering instead of alleviating them. The surgeons said that overtreatment occurred because it is easier to act than refrain from action, and that it was difficult to refuse the patients' wishes.

#### Respecting patients

The surgeons emphasized that the patients' right to decide their own treatment created ethical dilemmas. Not being allowed by the patients to operate where there are possibilities of full recovery or substantial improvement was a difficult dilemma for the surgeons, e.g. patients with cancer. Members of Jehovah's Witnesses who refuse blood transfusion create a dilemma if the operation entails a high risk of life-threatening blood loss. "In a way they force me to participate in a situation where I can risk losing a patient who I know I could easily have saved". Another dilemma arose when parents ask for ritual circumcision of baby boys.

Respecting patients' right to decide their own treatment meant that the surgeons felt responsible for informing the patients about their diseases, the risks and benefits of surgery and other medical treatments available, and for giving advice to help patients make the 'right' decision. "I cannot remove any parts of a person's body unless they are accountable; unless they themselves comprehend that it is necessary". Deciding the right amount of information to give the patients was not always easy, according to the surgeons. Assessing the accurate risk and benefits of an operation was experienced as difficult in each individual case. Also presenting the risks and benefits of undergoing or not undergoing treatment in a neutral and unbiased way could also be difficult. The surgeons said that patients with unquestioning faith in the surgeon may give their consent to surgery without having understood the consequences.

It was experienced as difficult to assess patients' understanding of the surgeons' information about diagnostics and treatment options. "It can sometimes be difficult to give enough information. Even if you spend a lot of time providing information, it is still not certain that the patients understand". The surgeons said it was difficult to perform operations when they were in doubt about patients' capacity to make informed decisions, e.g. dementia sufferers, or psychiatric patients with reduced mental capacity. "Patients suffering from dementia will experience any operation as an encroachment because they cannot take part in a discussion".

The surgeons complained about having no separate room where they could deliver sensitive information and bad news to patients and relatives. To use the corridor or other places where they could be overheard was experienced as not respecting the patients. "I think that the greatest problem in this hospital is that they [the administrators] do not comprehend that this is something we do almost every week with patients and relatives".

Patients seeking alternative treatment, medicines and drugs where the effects are uncertain and impossible to control was experienced as a dilemma for the surgeons. Although the surgeons respected the patients' right to decide their own treatment, they feared that some alternative treatment may harm the patients' standardized medical treatment. In some cases, patients with incurable cancer even have alternative operations in private clinics. "The patients may use all their savings paying for the treatment and then it turns out to be of no benefit to them". The surgeons said that their patients often seek advice about alternative medicine and they experienced a dilemma when they felt compelled to advice against alternative medicine as they feared that the patients might lose hope.

#### Meeting patients' expectations

The surgeons revealed that they are willing to go to considerable lengths in order to fulfil their patients' expectations. They experienced that many patients have "unrealistic" expectations of surgery and do not accept that surgery cannot do everything to relieve their ailments and impairments. "Sometimes they have pains or other torments that they expect to disappear after the operation. And they cannot see the connection between their torments and what they can do themselves to get better".

The surgeons told that patients have a strong belief in the health care system and expect, and even demand, to have the best available diagnostics and treatments. The surgeons emphasized that they have to trust the patient and believe in their complaints. If, however, they found that the clinical picture did not correspond to the patients' complaints, then they experienced the dilemma of how far they should go to examine the patients to rule out possible serious diseases. They said this is also a question of the use of limited resources, as extensive examinations of one patient will reduce resources available for other patients.

The surgeons emphasized that when patients have expectations they cannot fulfil, they tried to meet the patients part-way by providing information and education. If the patients' demands exceeded what surgeons found medically advisable and justifiable, they negotiated with the patients to find workable solutions. They referred patients to other physicians in extreme cases. Refusing patients' demands for narcotics, medical service, sick leave, social insurance etc. were experienced as dilemmas by surgeons who are trained to believe in the patient's complaints. Some surgeons said they have been exposed to physical and verbal threats by patients.

### Resolving differences in opinions

#### Superior colleagues

The interviews revealed that even though surgeons had different opinions, they usually reached agreement about the most suitable treatment for the patients. The surgeons experienced a dilemma whether they should act against or give in to the chief surgeons. "Sometimes you are asked to do something you do not think is right. You are for instance asked to do things in a particular way, and then you think that it is difficult to go against the decisions of those who are more experienced. It does not need to be anything strictly right or wrong, but minor things, like you would have chosen another type of drug". The surgeons felt they had to make decisions on their own on evening and night duty, because they found it difficult to wake the chief surgeon for advice. "Perhaps I make more decisions on my own then, because I feel that I have to sort it out on my own".

#### Colleagues from other departments

The surgeons experienced a dilemma if specialists from other departments ordered operations which the surgeons did not approve of themselves, for instance the feeding of severely ill, old or terminal patients with PEG-tubes. If the patients were not able to express what they want, the surgeons found it difficult to go against the specialists issuing the orders.

#### Incompetent colleagues

Correcting a colleague who is not competent or who has unacceptable behavior in relation to patients, was felt as a personal and a professional dilemma for the surgeons, who said that they felt responsible for both their patients and their colleagues. They said that the standard of surgical performance is high and it is difficult to determine fairly and objectively whether a colleague is performing adequately or not. "On the one hand you realize that surgery is complicated, and you cannot blame people for a single decision or a single action that is wrong". The chief surgeons in particular said they felt responsible towards the patients and society, to stop incompetent colleagues from practicing surgery.

### Society

#### Local limited resources

The interviews revealed that surgeons had to consider prioritization of limited resources in their decision-making. The amount of resources spent on one patient affected other patients on the waiting lists for operations. The dilemma of making the right prioritization was always present, according to the surgeons. They also mentioned that it was an ethical dilemma whether economical considerations should influence decisions about withholding or withdrawing medical treatment and if so, how much. The surgeons had to prioritize between patients when all the beds in the intensive care unit were occupied. Intensive treatment of seriously ill patients implies spending a lot of resources on one patient over time. They said that by prolonging the intensive treatment of very old patients, other patients in need may not receive treatment, operations are delayed and patients waiting for operations risk having complications because of the delay. Time shortage may also harm the quality of medical care.

Long waiting lists for surgical treatment are a problem in the health care system. The surgeons were concerned that some patients receive treatment too late because they are omitted from the waiting lists for some reason or other, mainly because of the breakdown of routines.

#### Laws and regulations

The surgeons were upset because they are legally responsible for informing the patients about 'DNR' orders '(do not resuscitate') and document it in the patients' medical records. Although they stressed that every patient has a right to be given sufficient information about medical examination and treatment according to legalisation on patients' rights, they felt that this regulation was inhuman and removed the patients' spark of hope that enable them to fight the disease. They therefore said that they placed consideration for patients before laws and regulations. The surgeons also mentioned the regulation that parents have no economic right to be with their sick children unless they are dying, which they thought was preposterous, and they therefore tried to bend the rules.

In the recent years the surgeons have been ordered to document their activities in a code system based on diagnosis-related groups (DRGs). The patients' diagnoses and the sequences in which they are coded determine the amount of government reimbursement the hospital receives. The surgeons felt pressure to report the patients' diagnoses in such a way that reimbursement to the hospital increased, but stressed that they document in agreement with their assessment of the patients' ailments. They feared that the DRG system would influence their way of working in a negative way.

#### Global limited resources

The surgeons expressed a global perspective on their work, realizing that they have more available resources at their disposal than physicians in other parts of the world, and that the global distribution of health care resources is unfair. "For instance, in our part of the world we are using enormous resources in trying to help an 85-year-old to have one or two more years to live, while in other parts of the world they are striving to keep children alive". From a global perspective, the surgeons experienced a dilemma in that people in our part of the world do no realize that both local and global resources are limited, but insist having the best treatment whatever the cost. This dilemma was especially pronounced when they experienced increasing expectations and pressure from patients and society to do even more despite limited and unequally distributed global resources. However, they found it gratifying having enough resources "to do a conscientious and decent job" and "to be of use to society" under the present circumstances. They appreciated the recognition they received from patients and relatives.

## Discussion

The surgeons in this study narrated about the many and complicated ethical dilemmas connected to the treatment of patients, differences of opinions between colleagues and prioritization of limited local and global resources. The main focus in the narratives was on ethical dilemmas concerning the patients' treatment and quality of life, patients' right to decide and meeting patients' expectations. Trying to find the ethically correct action that benefited their patients most pervaded the surgeons' narratives. Consideration for the patients was salient even when they told about resolving differences in opinions with colleagues and conforming to the laws and regulations in society. It seems that the ethical dilemmas the surgeons experienced originated mainly out of regard for their patients, and this was equally important in resolving the dilemmas, for instance by bending social laws and regulations in the patients' and relatives' favor.

The surgeons' ethical awareness is not highlighted in previous research. On the contrary, it is suggested that surgeons until recently have taken less interest in ethics than their colleagues in other medical areas [8,10,36-39]. The surgeons in this study however, reflected over and related many different ethical dilemmas in their everyday practice. They are aware of the many ethical dilemmas in their practice and are dedicated to the best possible outcome and care for each patient using their scientific knowledge and practical wisdom. Thus it seems reasonable to suggest that they take both their professional and ethical responsibility seriously. To be morally committed to patients is to be aware of the responsibility inherent in the patient-physician relationship. This ethical responsibility arises from the ethical demands with which they are faced in their meetings with patients. It is necessary for every person to act in a way that is determined by our understanding of a situation and related to the particular risk to which we are subjected, according to the Danish philosopher Løgstrup [40]. Our understanding of the situation presents us with a choice which in turn demands that we act and give of ourselves and thereby contribute to the realization of one or more other individuals' possibilities in life, which is at our disposal in the given situation. The physicians' challenge is to be open to the ethical appeal without being destroyed by an overwhelming responsibility [41].

The surgeons in this study showed a strong dedication to their patients' well-being, treatment and care in their relationships with patients [24] as well as when they face the ethical dilemmas in clinical practice. Other studies have found that physicians in ethically difficult situations were preoccupied with making decisions based on factual, scientific knowledge and that this knowledge was considered essential in order to give the patients qualitatively good treatment [1,28,30]. In this study, however, the surgeons did not mention scientific knowledge as a resource in resolving ethical dilemmas. They seemed to be preoccupied with performing surgery in a way that led to something beneficial for their patients. In the other paper based on this study we have described the surgeons' involvement in their patients' destinies and the way this affected their own lives [24]. This study seems to contradict the myth of surgeons as distant and indifferent technicians.

Patients expect to benefit from surgical care. They trust surgeons to exercise their knowledge and skills to the best of their ability, and assume that they will take all reasonable steps to ensure a favorable outcome. In this respect the surgeons and patients and relatives interests in most instances seems to correspond. Physicians usually want to respond to patients' expectations by taking action [42]. The surgeons in this study said they felt obliged to exert themselves to help patients for whom they are responsible and thereby meet their expectations. Giving the patient a chance means that the surgeons take personal and professional risks because they can never know the outcome of their decisions, thus the risk of overtreatment [18,24,27,32,43]. The surgeons deliberations can be understood in an Aristotelian perspective as an expression of clinical phronesis; a search for the middle course of action, which lies somewhere between the excess involved in "doing everything possible" or "doing nothing at all" [44,45]. The role of the physician is said to require both the ability and courage to act in uncertain and high-risk situations where the outcomes may be marginal [46]. It may seem that both physicians and patients want to force medicine to the edge of what medicine can accomplish and patients can endure.

Physicians assume personal responsibility for the way they manage their patients and do so; on the basis of their own clinical experience [42]. The role of a surgeon in our culture is filled with high expectations. Meeting patients' and relatives' expectations was experienced as a dilemma for the surgeons because these expectations in many instances were experienced as unrealistic and unlimited. Many other authors have found that physicians working in different parts of the health care system experience greater expectations from patients, relatives and the media than they can fulfil [3,11,15,18,20,23,27,47-49]. Pahuus [50] writes that when our expectations are without limits and countless, we tend to assume the object of our expectation as being perfect. Unlike the patients, the surgeons also have knowledge and experiences of the limitation of surgery [24]. Furthermore, it belongs to the surgeons' ethical responsibility to restrict their intervention to the cases where they have reasonable prospects to succeed [41]. This ethical dilemma facing surgeons will probably not decrease in the future due to the rising cost of modern medicine. As the surgeons' knowledge and skills exceed the available resources, they will experience the ethical dilemma of distributing health care to a greater degree [3,10,17].

The physician's role is said to have changed from paternalism to respecting patients' autonomy [15,22]. Several studies have found that physicians emphasize the importance of respecting the patients and acknowledging their autonomy and authority to decide their own treatment and judge their own quality of life [15,32,47,49]. Falkum & Førde [22] found in their study that surgeons had the lowest scores for valuing patients' autonomy, and tended to decide for their patients without wasting time on information and dialogue. In this study, the surgeons emphasized the importance of showing respect for patients' rights to decide to choose to undergo surgical treatment or not, and the importance of providing adequate information in a way that the patients understood. Studies show conflicting results as to whether the patients' wish to participate in decisions concerning their own treatments or not. Some studies seem to confirm the experiences of the surgeons in this study; that patients want to be well informed, they want more information then they usually get, but often prefer that the final decisions are made by physicians [15,51].

The surgeons in this study seemed to be content in performing their profession and of being in a position where they are able to cure people and alleviate suffering. They appreciated the recognition they received from patients and relatives. This recognition is based on their ability to act in a correct way as well as their attitude in their relationships with patients. It helped the surgeons that they remembered cases were they succeed against all odds although they found it difficult to withhold treatment with uncertain outcome. It is reasonable to suggest that the recognition they get from patients and especially from patients, who survive against all odds, and their relatives, gives them a social confirmation which is important for them [24,33]. This confirmation tells them that they are successful and proficient surgeons.

The surgeons felt personal responsibility for the treatment and care of their patients. They said that this responsibility is sometimes experienced as a heavy burden. According to Henriksen and Vetlesen [52] being responsible for another person's well being ought to be felt as a heavy burden in order to count as moral responsibility. In this study, the surgeons seem to accept the professional and moral challenges in their work in spite of their experiences with many different ethical dilemmas. They also seem content to be 'of use' both for their patients and not least, for society.

Everyone is vulnerable and it is reasonable to suggest that surgeons are especially vulnerable in their professional role as it often concerns questions about life and death, and issues that affect the patients' quality of life. Many authors have found that physicians accept their own vulnerability and fallibility when they are faced with difficult ethical dilemmas [15,26,28,33]. We found that the surgeons in this study seemed content to be of use both for their patients and for society. Thus, the gratifying aspects of their profession seemed to outweigh their vulnerability.

## Competing interests

The author(s) declare that they have no competing interests.

## Authors' contributions

KT participated in the design of the study, carried out the interviews, participated in the analysis and completed the manuscript.

AN read the interviews, participated in the analysis and helped to draft the manuscript.

VS participated in the design of the study, read the interviews, participated in the analysis and helped to draft the manuscript.

All authors have read and approved the final manuscript.

## Pre-publication history

The pre-publication history for this paper can be accessed here:


